# A Mechanistic Study of the Antiaging Effect of Raw-Milk Cheese Extracts

**DOI:** 10.3390/nu13030897

**Published:** 2021-03-10

**Authors:** Guillaume Cardin, Cyril Poupet, Muriel Bonnet, Philippe Veisseire, Isabelle Ripoche, Pierre Chalard, Anne Chauder, Etienne Saunier, Julien Priam, Stéphanie Bornes, Laurent Rios

**Affiliations:** 1 Université Clermont Auvergne, INRAE, VetAgro Sup, UMRF, F-15000 Aurillac, France; cyril.poupet@uca.fr (C.P.); muriel.bonnet@uca.fr (M.B.); philippe.veisseire@uca.fr (P.V.); stephanie.bornes@uca.fr (S.B.); laurent.rios@vetagro-sup.fr (L.R.); 2 Université Clermont Auvergne, CNRS, Clermont Auvergne INP, ICCF, F-63000 Clermont-Ferrand, France; isabelle.ripoche@sigma-clermont.fr (I.R.); pierre.chalard@sigma-clermont.fr (P.C.); 3 Dômes Pharma, ZAC de Champ Lamet, 3 Rue Andrée Citroën, F-63284 Pont-du-Château, France; a.chauder@domespharma.com (A.C.); e.saunier@domespharma.com (E.S.); j.priam@domespharma.com (J.P.)

**Keywords:** raw-milk cheese, *Caenorhabditis elegans*, longevity, oxidative stress, DAF-16, p38 MAPK

## Abstract

Many studies have highlighted the relationship between food and health status, with the aim of improving both disease prevention and life expectancy. Among the different food groups, fermented foods a have huge microbial biodiversity, making them an interesting source of metabolites that could exhibit health benefits. Our previous study highlighted the capacity of raw goat milk cheese, and some of the extracts recovered by the means of chemical fractionation, to increase the longevity of the nematode *Caenorhabditis elegans*. In this article, we pursued the investigation with a view toward understanding the biological mechanisms involved in this phenomenon. Using mutant nematode strains, we evaluated the implication of the insulin-like DAF-2/DAF-16 and the p38 MAPK pathways in the phenomenon of increased longevity and oxidative-stress resistance mechanisms. Our results demonstrated that freeze-dried raw goat milk cheese, and its extracts, induced the activation of the DAF-2/DAF-16 pathway, increasing longevity. Concerning oxidative-stress resistance, all the extracts increased the survival of the worms, but no evidence of the implication of both of the pathways was highlighted, except for the cheese-lipid extract that did seem to require both pathways to improve the survival rate. Simultaneously, the cheese-lipid extract and the dried extract W70, obtained with water, were able to reduce the reactive oxygen species (ROS) production in human leukocytes. This result is in good correlation with the results obtained with the nematode.

## 1. Introduction

In the last few years, many studies have highlighted the relationship between diet and health status with the aim of improving both disease prevention and life expectancy. Among the different food groups, fermented foods, which represent an important part of our diet, have a huge microbial biodiversity that makes them an interesting source of metabolites that could exhibit health benefits. Recent studies have demonstrated that fermented foods may exhibit various beneficial effects on health, such as a cardiovascular protective effect [1,2] or an antiproliferative activity in the field of cancer prevention [3]. In a previous study [4], we focused our interest on one particular fermented food, raw goat milk cheese. We highlighted the development of a new methodology allowing us to fractionate cheese, using chemical fractionation, and to highlight the effects of the whole cheese, as well as the resulting extracts, on the longevity of the nematode *Caenorhabditis elegans*. We demonstrated a pro-longevity effect of the freeze-dried cheese and of some of the extracts (a lipophilic extract, named cheese-lipid extract, and three different hydrophilic extracts, named W40, WF and W70) on an in vivo model, using the wild-type *C. elegans* N2 strain. The freeze-dried cheese presented the ability to increase the maximum lifespan by 63%. The cheese-lipid extract increased longevity up to 37%. The three hydrophilic extracts also increased the maximum lifespan, between 13% and 73%, depending on the concentration. Another notable particularity revealed by this assay was the percentage of the population remaining alive on the extracts after all the nematodes in the control group had died: freeze-dried cheese (13%), cheese-lipid extract (up to 5%) and hydrophilic extracts (between 4% and 16%).

In the present study, we pursued the investigation of the health benefits of raw goat milk cheese, and its metabolites, using the methodology developed in our previous study [4]. In order to better understand the mechanisms of the cheese extracts in increasing longevity, an exploration of the signaling pathways involved was performed using an in vivo *C. elegans* model. This model has shown to be efficient in many studies that evaluated the health impact of some plant extracts and microorganisms [5,6]. *C. elegans* was chosen for our studies because of its similarities with humans concerning the physiology of the intestinal cells [7] and the homology of many signaling pathways [8], that make it a relevant model for mechanistic studies. Many mutant strains are available, which allow us to characterize the implication of these signaling pathways in the effects of the extracts. In *C. elegans*, the aging process is modulated by highly conserved signaling pathways, such as the DAF-2/DAF-16 [8,9]. The transcription factor DAF-16 has been demonstrated to regulate downstream genes that influence longevity [10,11] and may be involved in the mechanism of action of the extracts in increasing life expectancy. The implication of this pathway will be investigated by using a mutant strain that did not express the transcription factor DAF-16.

With aging, age-related affections become ever more prevalent. Many processes are involved in these affections, such as the oxidative process in which the reactive oxygen species (ROS) are implicated. These compounds cause damage to lipids, proteins and DNA, which results in the death of the cell [9] and, in the end, of the organism. Moreover, during aging, the defense mechanisms of the worms are weakened [9], leaving the nematode more sensitive to the oxidative stress, which can be combined with an excessive production of ROS [12]. Consequently, the capacity of the extracts to improve the nematode survival rate on an oxidative medium was investigated in parallel with the longevity test. In keeping with the exploration of the effects of the extracts on oxidative stress, an in vitro study was conducted to measure the ability of the extracts to reduce the ROS production in human leukocytes and to correlate the results obtained with those in the nematode.

As cheese is a fermented food prepared from milk, a final investigation was performed in order to estimate the impact of the milk fermentation on the production of bioactive metabolites. To do so, the same fractionation as described in our previous work [4] was performed on the nonfermented milk in order to obtain raw goat milk extracts that were then evaluated with a longevity and survival assay on the oxidative medium.

## 2. Materials and Methods

### 2.1. Milk and Cheese Samples

Raw goat milk, freshly collected, and raw goat cheese (ripened for 20 days) were taken from a local producer (Chèvrerie des Oliviers, Saint-Georges sur Allier, France). The milk was concentrated under vacuum and freeze-dried (FreeZone Triad Freeze Dryer, Labconco Corporation, Kansas City, MS, USA). The resulting solid was crushed with mortar and pestle and the freeze-dried milk (FDM) was kept in a waterproof container at 4 °C. The cheese was cut into small slices, freeze-dried and crushed with mortar and pestle. The freeze-dried cheese (FDC) was kept in a waterproof container at 4 °C [4].

### 2.2. Reagents and Solvents

Five-fluoro-2′-deoxyuridine (FUdR), KH_2_PO_4_, amphotericin B (250 µg/mL), NaOH, agarose, cholesterol, CaCl_2_, NaCl, EDTA, RPMI, Na_2_HPO_4_, MgSO_4_, potassium phosphate buffer, NH_4_Cl, NaHCO_3_, phorbol myristate acetate (PMA), fetal bovine serum (FBS), gentamicin, glutamine and resazurin were bought from Sigma Aldrich (Saint Louis, MO, USA). lysogeny broth (LB, Miller’s Modification), peptone and agar were obtained from Conda (Madrid, Spain). Yeast extract was obtained from Fisher Scientific (Hampton, VA, USA). Dihydrorhodamine 123 (DHR 123) was purchased from Cayman Chemical Company (Ann Arbor, MI, USA). TRIzol was acquired from Ambion by life technologies (Carlsbad, CA, USA). The High-Capacity cDNA Archive kit was obtained from Applied Biosystems (Foster City, CA, USA). Rotor-Gene SYBR Green Mix was acquired from Qiagen GmbH (Hilden, Germany) and primers from Eurogentec (Seraing, Belgium).

### 2.3. Obtaining of Milk Extracts

**Figure 1:**
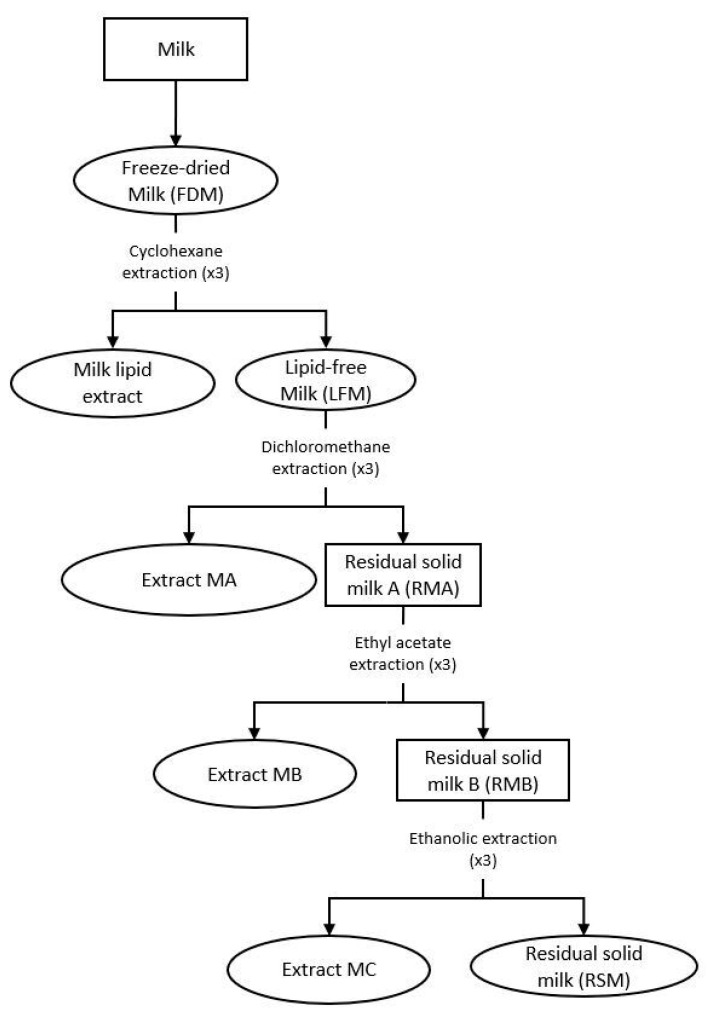
Preparation of the milk extracts. Ovals designate the milk extracts used for our biological studies (FDM (freeze-dried milk), milk lipid extract, LFM (lipid-free milk), and extracts MA (obtained with dichloromethane), MB (obtained with ethyl acetate), MC (obtained with absolute ethanol) and RSM (residual solid milk)).

An extraction procedure was performed on the freeze-dried milk (FDM) to recover the different extracts, as mentioned in our previous article [4]. The apolar extract of the milk metabolites was recovered by adding distilled cyclohexane (ratio 1/10 (*w*/*w*)) to FDM powder and mechanically agitated for 4 h. The solution was then filtered with Büchner and evaporated under vacuum. The resulting solid was dissolved in cyclohexane (ratio 1/10 (*w*/*v*)) before filtering again to eliminate the residue of the milk and evaporated under vacuum to obtain the dry extract. The milk matrix was exhausted by repeating the same procedure three times, under the same conditions at different times (4 h, 2 h and 1 h, respectively). The resulting dry extracts were combined to constitute the final extract, known as denominated milk lipid extract. The residual solid, which was retained by the filtration, was dried under vacuum and named lipid-free milk (LFM) (Figure 1).

The LFM was extracted using a chemical fractionation to recover most of the compounds, which resulted in three successive solid/liquid extractions, with an increase of the polarity of the solvent at each step: dichloromethane, ethyl acetate and absolute ethanol (Figure 1). For each solvent, the protocol used was the same as with cyclohexane, but with a 1/10 (*w*/*v*) ratio. The final dried extracts were denominated as extracts MA, MB and MC, respectively.

The residual solid from the absolute ethanol extraction was dried under vacuum and named residual solid milk (RSM). Each dried milk extract was kept in a waterproof container at −25 °C, under argon.

### 2.4. Obtaining of Cheese Extracts

The same cheese extracts were used in this study as in our previous work [4]. The cheese extracts were obtained from the goat cheese by successive chemical extractions as described in the previous study. Briefly, the same extraction procedure as for the milk was applied to the freeze-dried cheese (FDC) to obtain a cheese-lipid extract, which was extracted with cyclohexane, as well as lipid-free cheese. From this residue, extract A and residue RA were then obtained with dichloromethane. Next, extract B and residue RB were obtained from the residue RA with ethyl acetate. To finish, extract C and residue RC were obtained from the residue RB with absolute ethanol. A fifth extraction was performed on the residual solid RC recovered from the ethanolic extraction to obtain yet another extract. HPLC-grade water was added to this solid (ratio of 1/10 (*w*/*v*)) and the mixture was mechanically agitated for 1 h at 40 °C. The mixture was then centrifuged (8000 rpm, 15 min; Avanti J26S XPI, Beckman Coulter, Brea, CA, USA) and the supernatant was concentrated under vacuum, filtered to eliminate the residue of cheese and evaporated. Finally, the resulting solid was dried under vacuum. The cheese matrix was exhausted by repeating the same procedure three times, under the same conditions. The resulting dry extracts were combined to constitute the final extract, ground with mortar and pestle and denominated extract WF.

Two additional extracts were obtained by performing a water extraction on the lipid-free cheese, at two different temperatures (40 °C and 70 °C). The extraction procedure was the same as that for the extract WF described above, with the exception of the temperature. The resulting extracts were named W40 (extracted at 40 °C) and W70 (extracted at 70 °C).

Each dried cheese extract was kept in a waterproof container at −25 °C, under argon. The following experiments were performed using the FDC, the cheese-lipid extract and the extracts W40, W70 and WF, all of which presented a beneficial effect on the worms’ lifespan in our previous study.

### 2.5. Microbial Strains, Growth Conditions and Heat-Killed Preparation

The *Escherichia coli* strain OP50 was provided by the *Caenorhabditis* Genetics Center (Minneapolis, MN, USA) and used as a food source during the worms’ maintenance. *E. coli* OP50 was grown in lysogeny broth medium at 37 °C overnight. The microbial suspension was centrifuged (15 min, 4000 rpm; Rotofix 32A, Hettich Zentrifugen, Tuttlingen, Germany) and washed with M9 buffer (per L: 3 g of KH_2_PO_4_; 6 g of Na_2_HPO_4_; 5 g of NaCl; 1 mL of 1 M MgSO_4_). The microbial suspension was adjusted to obtain a 100 mg/mL final concentration. During the experiments the worms were fed with heat-killed (HK) *E. coli* OP50. *E. coli* (100 mg/mL) (from the resulting suspension) was heat-killed at 70 °C for 1 h and the solution was kept at 4 °C until use.

### 2.6. C. elegans Maintenance

The *Caenorhabditis elegans* N2 (wild-type), and the different mutants TJ356 (*daf-16p::daf-16a*/*b::GFP* + *rol-6(su1006)*), GR1307 (*daf-16(mgDf50)*) and IG10 (*tol-1(nr2033)*) strains were acquired from the *Caenorhabditis* Genetics Center. The nematodes were cultured at 20 °C on nematode growth medium (NGM) plates (per L: 3 g of NaCl; 2.5 g of peptone; 17 g of agar; 5 mg of cholesterol; 1 mM of CaCl_2_; 1 mM of MgSO_4_; 25 mL of 1 M potassium phosphate buffer at pH 6), supplemented with yeast extract (4 g/L) (NGMY) and seeded with live *E. coli* OP50 [13,14,15].

### 2.7. Synchronisation of Wild-Type C. elegans and Mutant Strains

In order to avoid any variation in the results due to the age differences in the population, a synchronization of the worms was performed. Gravid worms and eggs were collected from NGMY plates and washed off using M9 buffer before centrifuging (2 min, 1500 rpm; Rotofix 32A, Hettich Zentrifugen, Tuttlingen, Germany). Five milliliters of worm bleach (2.5 mL of M9 buffer, 1.5 mL of bleach, 1 mL of sodium hydroxide 5 M) were added to the pellet and vigorously shaken until adult worm body disruption. Forty milliliters of M9 buffer were then introduced to block the effect of the worm bleach. The egg suspension was centrifuged (2 min, 1500 rpm) and washed twice with 20 mL of M9 buffer. The isolated eggs hatched at 25 °C for 24 h in 20 mL of M9 buffer, under slow agitation. The resulting L1 larvae were then transferred onto NGMY plates, seeded with live *E. coli* OP50 as a food source and maintained at 20 °C until they reached the L4/young adult stage [13,14,15].

### 2.8. Caenorhabditis elegans Longevity Assay Incubated with Dried Milk Extracts

The effect of the dried milk extracts on the life expectancy of the worms was evaluated by performing a longevity assay using the wild-type *C. elegans* N2 strain. An agar medium (per L: 3 g of NaCl and 6 g of agarose) was prepared, stored at 40 °C and split into aliquots that were individually supplemented with the dried milk extracts at the suitable concentration, according to the physicochemical properties of the extracts (Table 1). The supplemented aliquot was then poured, at 40 °C, into a 24-well plate with 0.12 mM of FUdR. The aliquot was also supplemented with amphotericin B (final concentration of 16 µg/mL), in the case of the lipid-free milk (LFM), to prevent any significant fungal development, which is commonly observed with this extract [16]. The presence or absence of the antifungal established two control conditions: with and without amphotericin B. To densify the agar, the plates were immediately moved onto ice after pouring, and kept at 4 °C until being used. Once adult, worms were incubated on a supplemented agar medium (or agar medium for the control condition) with ~15 worms per well, with HK *E. coli* OP50 as a food source, and kept at 20 °C for the duration of the assay. To avoid starvation, food was added every 3 days in the wells (20 µL of 100 mg/mL suspension). Nematodes were observed daily and were considered dead when they did not respond to a gentle mechanical stimulation. The effect of the LFM was evaluated in comparison with the control condition with amphotericin B and food (CC2), whereas the effect of the other extracts was evaluated in comparison with the control condition with food only (CC1). This assay was performed as at least three independent experiments containing three wells per condition and conducted simultaneously with the control conditions. Complementary information was taken into account to determine the effects of the extracts: the observation of the relative position of the curves, the value of the mean and maximum lifespan and the percentage of the population that was still alive on the supplemented medium when the worms in the control condition were all dead [4].

### 2.9. Longevity Assay of DAF-16 Loss of Function Mutant (GR1307 Strain) Incubated with Dried Cheese Extracts

In order to determine the implication of DAF-16 in extending the longevity of the worms incubated with dried cheese extracts, a longevity assay was conducted with the *C. elegans* GR1307 strain (DAF-16 loss-of-function). To prepare the supplemented agar medium with the cheese extracts, the same protocol described above was applied. The dried cheese extracts were used at 0.5% and 1% concentrations (*w*/*v*) for supplementing the medium. The aliquots were also supplemented with amphotericin B (1.6 µg/mL) in the case of the freeze-dried cheese (FDC) and the cheese-lipid extract, to prevent any significant fungal development. The effects of the FDC and the cheese-lipid extract were evaluated in comparison with the control condition with amphotericin B and food (CC2), whereas the effects of the extracts W40, W70 and WF were evaluated in comparison with the control condition with food only (CC1). This assay was performed as at least four independent experiments containing three wells per condition and conducted simultaneously with the control conditions.

### 2.10. Cellular Localisation of DAF-16::GFP

In order to study the biological activity of dried cheese extracts, an experiment was performed using transgenic TJ356 worms (DAF-16::GFP). The nuclear localization of the transcription factor was determined thanks to fluorescence, as described by Poupet et al. [14]. The same agar medium as described for the longevity assay was prepared, cooled down to 40 °C and supplemented with 5 mg/L of cholesterol, 1 mM of CaCl_2_, 1 mM of MgSO_4_ and 1 M of potassium phosphate buffer at pH 6 (25 mL for 1 L of medium). The medium was then split into aliquot, and individually supplemented with cheese extracts at 1% (*w*/*v*) and poured into a 24-well plate before being immediately transferred onto ice to densify the agar, and stored at 4 °C until use. Once adult, worms were incubated on a cheese-extract agar plate for 2 h and 4 h at 20 °C, with food (heat-killed *E. coli* OP50). The translocation of DAF-16::GFP was scored by assaying the presence of the GFP accumulation in the *C. elegans* cell nuclei, using a 40× magnification fluorescence microscope (Evos FL, Invitrogen, Carlsbad, CA, USA) [17].

### 2.11. Survival of the Worms on the Oxidative Medium

The experiment was performed to determine the potential antioxidant activity of dried milk and cheese extracts as a preventive measure. This study was performed with the wild-type *C. elegans* N2 strain for the milk and cheese extracts. The experiment was also performed with the GR1307 strain (DAF-16 loss-of-function) and the IG10 strain (TOL-1 loss-of-function), but only for the dried cheese extracts. This assay was performed according to Grompone et al. (2012) [18], with some modifications. The same agar medium as described for the longevity assay was prepared and supplemented as described above with dried milk or cheese extracts at 1% (*w*/*v*). The aliquots were supplemented with food (40 µL of HK *E. coli* OP50 suspension at 100 mg/mL) for each condition, before being poured, at 40 °C, into a 24-well plate with 0.12 mM of FUdR. The aliquots were also supplemented with amphotericin B (1.6 µg/mL) in the case of the lipid-free milk (LFM), freeze-dried cheese (FDC) and cheese-lipid extract to prevent any significant fungal development. As described above, two control conditions were used during the assay: with and without amphotericin B. After pouring, the plates were immediately transferred onto ice to densify the agar, and stored at 4 °C until use. Once adult, worms were incubated on a supplemented agar medium (or agar medium for the control condition) with ~50 worms per well, and the worms were incubated for 5 days at 20 °C. After incubation, the worms were transferred onto an agar medium with or without hydrogen peroxide (3 mM in the medium). After 3 h 30 min of contact, the worm survival rate τ was scored and expressed with the following formula:

(1) $$\tau =\frac{{\left(\frac{n_{\mathrm{living}\mathrm{worms}\;\mathrm{at}\;t=3h30}}{n_{\mathrm{living}\;\mathrm{worms}\;\mathrm{at}\;t=0h}}\right)}_{{\mathrm{medium}\;\mathrm{with}\;H}_{2}O_{2}}}{{\left(\frac{n_{\mathrm{living}\;\mathrm{worms}\;\mathrm{at}\;t=3h30}}{n_{\mathrm{living}\;\mathrm{worms}\;\mathrm{at}\;t=0h}}\right)}_{{\mathrm{medium}\;\mathrm{without}\;H}_{2}O_{2}}},$$

A worm was considered as dead when it did not respond to a mechanical stimulus. The effect of the LFM, FDC and the cheese-lipid extract was evaluated in comparison with their respective control condition with amphotericin B and food (CC2) whereas the effect of the other extracts was evaluated in comparison with their respective control condition with food only (CC1). This assay was performed as at least four independent experiments containing three wells per condition and conducted simultaneously with the control conditions.

### 2.12. Determination of the Expression of Gene of Interest

#### 2.12.1. Incubation of the Worms

In order to determine the expression of the gene of interest (GOI), an RNA isolation and RT-quantitative PCR were performed with the wild-type *C. elegans* N2 strain. The experiment was conducted in order to evaluate the gene expression at two incubation times as determined in the longevity assay performed in our previous study [4]: at 3 days (start of the decrease in the population during the longevity assay) and at 10 days (mean lifespan of the control population during longevity assay). The worms were incubated on the medium supplemented with the cheese extracts at 1% (*w*/*v*) as described for the longevity assay, in 55 mm diameter Petri dishes (1 per replicate and per time). Once adult, worms were incubated on a supplemented agar medium (or agar medium for the control condition) with ~500 worms per well (3 days) or ~1000 worms (10 days), provided with the necessary amount of food for each time and kept at 20 °C. The freeze-dried cheese (FDC) and the cheese-lipid extract were compared to the control condition with amphotericin B and food (CC2), whereas the extracts W40, W70 and WF were compared to the control condition with food only (CC1). This assay was performed as at least three independent experiments.

#### 2.12.2. RNA Isolation and RT-Quantitative PCR

The RNA isolation and RT-quantitative PCR were adapted from Poupet et al. [19]. After a 3- or 10 day-incubation period, the worms were collected with M9 buffer. The total RNA was extracted by adding 500 µL of TRIzol reagent. The worms were disrupted by using a Precellys (Bertin instruments, Montigny-le-Bretonneux, France) and glass beads (PowerBead Tubes Glass 0.1 mm, Mo Bio Laboratories, Carlsbad, CA, USA). The beads were removed by centrifugation at 14,000 rpm for 1 min (Eppendorf^®^ 5415D, Hamburg, Germany), and 100 µL of chloroform was added to the supernatant. The tubes were vortexed for 30 s and incubated at room temperature for 3 min. The tubes were then centrifuged (12,000 rpm, 15 min, 4 °C) and the phenolic phase was removed. The aqueous phase was treated again with chloroform. The RNA was precipitated in the second aqueous phase by adding 250 µL of isopropanol. The tubes were incubated at room temperature for 4 min before centrifugation (12,000 rpm, 10 min, 4 °C). The supernatant was discarded, and the pellet was washed with 1000 µL of 70% ethanol. The supernatant was discarded after centrifugation (14,000 rpm, 5 min, 4 °C) and the pellet was dissolved into 20 µL of RNase-free water. Then, 2 µg of RNA was reverse-transcribed using the High-Capacity cDNA Archive kit, according to the manufacturer’s instructions. For real-time qPCR assay, each reaction contained 2.5 µL of cDNA, 6.25 µL of Rotor-Gene SYBR Green Mix, 1.25 µL of 10 µM primers (Table 2) and 1.25 µL of RNase-free water. All samples were run in triplicate. Rotor-Gene Q Series Software (Qiagen GmbH, Hilden, Germany) was used for the analysis. In our study, one reference gene, Y45F10D.4, was used in all of the experimental groups. The quantification of GOI expression (E_GOI_) was performed according to Equation (2) [20]:

(2) $$\mathrm{EGOI}=\;\frac{{\left(\mathrm{GOI}\;\mathrm{efficiency}\right)}^{\Delta {\mathrm{Ct}}_{\mathrm{GOI}}}}{{\left(Y45F10D.4\;\mathrm{efficiency}\right)}^{\Delta {\mathrm{Ct}}_{Y45F10D.4}}},$$

### 2.13. Leukocyte Viability

Blood was collected from healthy human volunteers (*n* = 22; Etablissement Français du Sang, EFS, Clermont-Ferrand, France). Donors gave their written informed consent for the use of blood samples for research purposes under EFS contract n°16-21-62 (in accordance with the following articles: L1222-1, L1222-8, L1243-4 and R1243-61 of the French Public Health Code). The whole-blood leukocytes were obtained by hemolytic shock using ammonium chloride solution (NH_4_Cl, 155 μM; NaHCO_3_ 12 μM, EDTA 0.01 μM). The leukocytes were then washed with RPMI, centrifuged (400× *g*, 10 min) and resuspended in RPMI. The cell preparations were adjusted to 10^6^ cells/mL with supplemented RPMI (FBS 10%, gentamicin 50 μg/mL and glutamine 2 mM). The cells were then placed in 96-well polystyrene plates (Cell Wells™, Corning, New-York, NY, USA), incubated with the dried extracts WF, W40, W70 or the dried cheese-lipid extract at 0, 10, 50, 100 or 200 µg/mL, PMA (1 µM) and resazurin (25 µg/mL). The extract WF was filtered at 0.22 µM to avoid any significant fungal development. Fluorescence (excitation/emission: 544/590 nm) was recorded every 30 min for 2 h using the Fluoroskan Ascent FL^®^ apparatus (ThermoFisher Scientific, Illkirch, France).

### 2.14. Kinetics of ROS Production by Leukocytes

Blood was collected from healthy human volunteers (*n* = 22). The whole-blood leukocyte preparations were obtained and adjusted as previously described. The cells were placed in 96-well polystyrene plates, incubated with the dried extracts WF, W40, W70 or the dried cheese-lipid extract at 0, 10, 50, 100 or 200 µg/mL, and dihydrorhodamine 123 (DHR 123, 1 μM), and stimulated by 1 µM PMA for 120 min to increase the ROS production. The extract WF was filtered at 0.22 µM to avoid any significant fungal development. The fluorescence intensity of rhodamine 123, which is the reduced form of dihydrorhodamine 123 oxidation by ROS, was recorded every 5 min for 120 min (excitation/emission: 485/538 nm) using the Fluoroskan Ascent FL^®^ apparatus.

### 2.15. Statistical Analysis

Results of lifespan experiments were examined by using the Kaplan–Meier method, and compared among group scoring for significance using the log-rank test with R software version 3.6.0. The differences between conditions, in the survival assay on the oxidative medium, the qPCR analysis, the cellular viability assay and the ROS production assay, were determined by using the Kruskal–Wallis test followed by an Uncorrected Dunn’s test using GraphPad Prism version 8.2.1 for Windows (GraphPad Software, La Jolla, CA, USA). Differences were considered statistically significant if *p*-value ≤ 0.05.

## 3. Results

### 3.1. Implication of DAF-16 in the Capacity of the Extracts to Induce an Increase in Longevity

In order to determine the implication of the transcription factor DAF-16 (involved in the longevity phenomenon) in the mechanisms of action of the dried cheese extracts (freeze-dried cheese (FDC), cheese-lipid extract, and WF, W40 and W70), a longevity assay was carried out using the GR1307 mutant strain that did not produce the protein. If the transcription factor DAF-16 was required, the extracts would no longer be able to induce an increase in the lifespan. In this regard, no variation of the beneficial effect of the cheese extracts should be noted.

**Figure 2:**
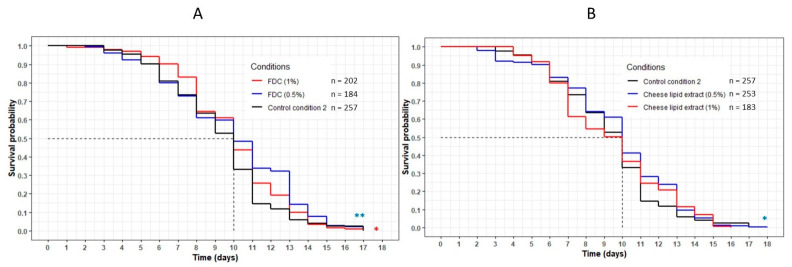
Influence of the FDC (freeze-dried cheese) (**A**) and the cheese-lipid extract (**B**) on the lifespan of *C. elegans* GR1307 strain. The worms were incubated on the medium supplemented with the dried extracts at day 0 and regularly fed with HK *E. coli* OP50. The conditions were considered significantly different when the *p*-value was lower than 0.05 (*) or 0.01 (**) (log-rank test). The asterisks next to the curves represent the differences with the control condition CC2. The asterisks next to the legend represent the differences between the extracts.

The worms incubated with the FDC did not show any variation in longevity compared with the control CC2 condition (Figure 2A). The mean and maximum lifespans were identical between the control condition and both concentrations of FDC (Table 3). However, the significant difference observed between the curves representing the FDC and the CC2 (*p* = 0.001 and *p* = 0.03 for 0.5% and 1% concentration, respectively) suggests that the FDC was responsible for a beneficial effect on the worms, allowing a larger part of the population to remain alive for a given amount of time compared with the CC2.

The cheese-lipid extract showed a variation between the two concentrations. An increase in longevity was observed for the worms incubated on the extract at 0.5%, with a significant difference between this curve and the CC2 curve (*p* = 0.02) (Figure 2B). The maximum lifespan was also higher, increasing from 17 (CC2) to 18 (+6%) days (Table 3). At 1%, the beneficial effects of the extract were no longer significant (*p* = 0.5).

**Figure 3:**
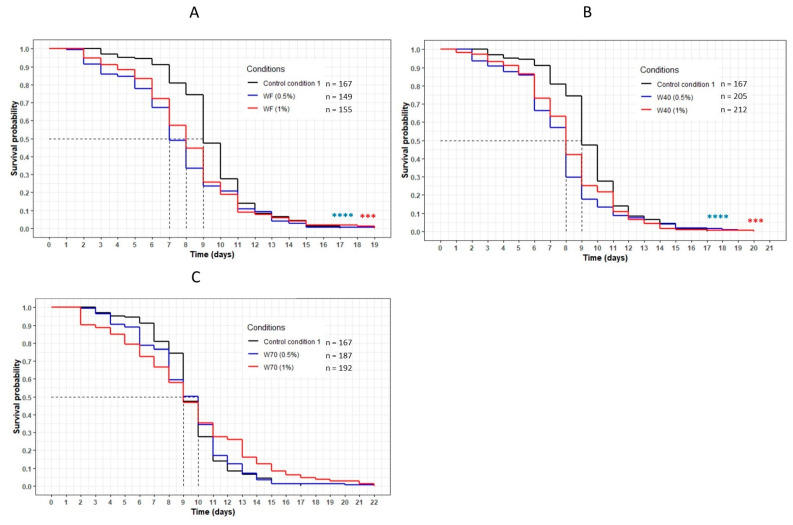
The influence of the aqueous extracts WF (**A**), W40 (**B**) and W70 (**C**) on the *C. elegans* GR1307 strain lifespan. The worms were incubated on the medium supplemented with the dried extracts at day 0 and regularly fed with HK *E. coli* OP50. The conditions were considered significantly different when the *p*-value was lower than 0.001 (***) or 0.0001 (****) (log-rank test). The asterisks next to the curves represent the differences with the control condition CC1. The asterisks next to the legend represent the differences between the extracts.

The worms incubated with the dried aqueous cheese extracts WF and W40 had a significant decrease in their lifespan compared with the control CC1 (*p* < 0.001) (Figure 3A,B). The curves representing these extracts were significantly below the CC1 curve, with a decrease in the mean lifespan from 9 (CC1) to 8 days (WF at 1%, W40 at 0.5% and 1%). Concerning WF at 0.5%, the mean lifespan decreased from 9 to 7 days (Table 4). However, the maximum lifespan was higher for all conditions, with an increase of between 12% and 18% compared with CC1. Concerning the extract W70, no variation in lifespan was observed in comparison with CC1. The evolution of the curve was similar to that of the control condition for both concentrations (Figure 3C). An increase in the maximum lifespan was noted for a small proportion of the population of the worms (up to 5% of the population).

**Figure 4:**
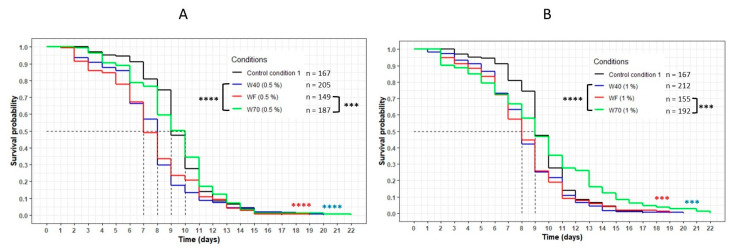
Comparison of the effect of the aqueous extracts WF, W40 and W70 at 0.5% (**A**) and 1% (**B**) concentration on the *C. elegans* GR1307 strain lifespan. The worms were incubated on the medium supplemented with the dried extracts obtained with water at day 0 and regularly fed with HK *E. coli* OP50. The conditions were considered significantly different when the *p*-value was lower than 0.001 (***) or 0.0001 (****) (log-rank test). The asterisks next to the curves represent the differences with the control condition CC1. The asterisks next to the legend represent the differences between the extracts.

This result was reinforced by the comparison of the three extracts WF, W40 and W70 at the same concentration (Figure 4). For both concentrations, the curve representing W70 was significantly above the other curves corresponding to the extracts WF and W40 (*p* < 0.0001 with W40; *p* = 0.0001 with WF). These results confirmed that the transcription factor DAF-16 was required for the FDC, the cheese-lipid extract and the extracts WF, W40 and W70 in order to increase the lifespan of the nematode *C. elegans* significantly.

### 3.2. Cellular Localisation of DAF-16::GFP

**Figure 5:**
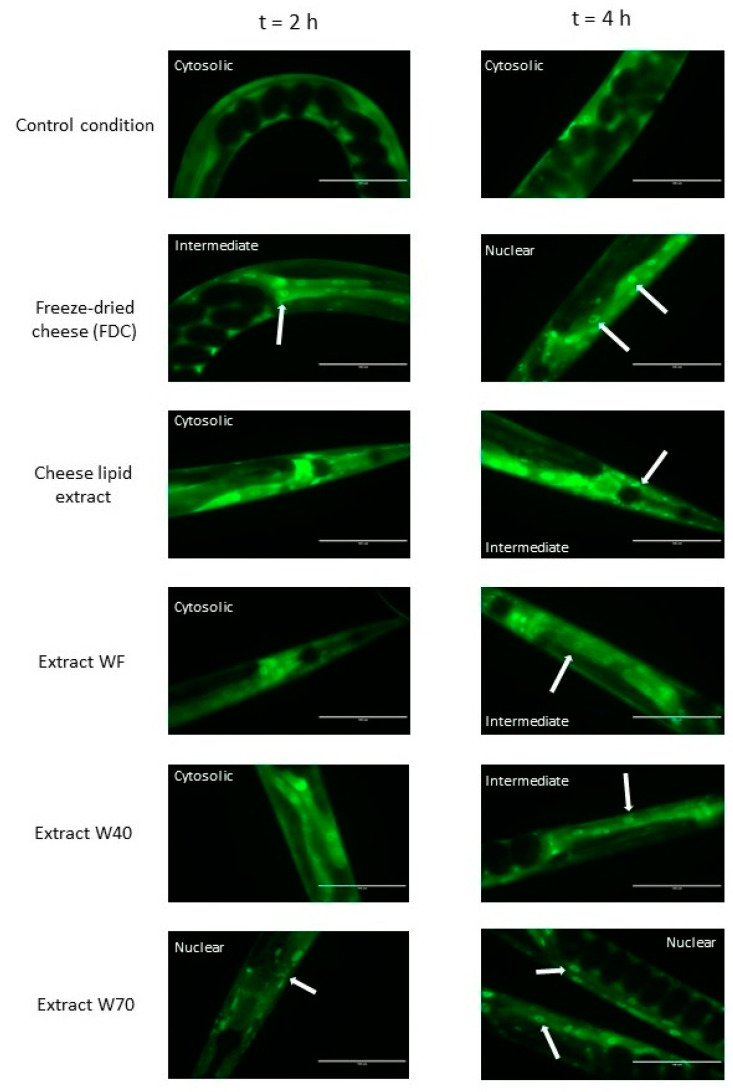
The effects of the FDC (freeze-dried cheese) and the extracts on DAF-16 cellular localization in *C. elegans* transgenic strain TJ356 expressing DAF-16::GFP, after 2 h and 4 h of incubation on the supplemented medium. The arrows indicate the accumulation of the transcription factor in the nuclei. Scale bar: 100 µm.

The nuclear translocation of the DAF-16/FOXO transcription factor was examined using the *C. elegans* TJ356 strain (which constitutively expresses the fusion protein DAF-16::GFP) (Figure 5). The localization of DAF-16 in the cells of the worms was established by fluorescence to determine if this signaling pathway was involved in the effects of the extracts. Figure 5 shows that the freeze-dried cheese (FDC) and the extract W70 were the only extracts that tended to induce a translocation of the transcription factor into the nuclei after 2 h of incubation. At 4 h, each extract tended to induce a translocation of DAF-16 into the nuclei. The control condition did not show any variation in DAF-16 cellular localization for the duration of the assay, suggesting that the translocation observed was induced by all of the extracts. These observations reinforced the results obtained from the longevity assays, suggesting that the transcription factor DAF-16 was involved in the mechanisms of action of the extracts.

### 3.3. Effect of the Dried Cheese Extracts on the Survival Rate of the Wild-Type C. elegans N2 Strain on the Oxidative Medium

**Figure 6:**
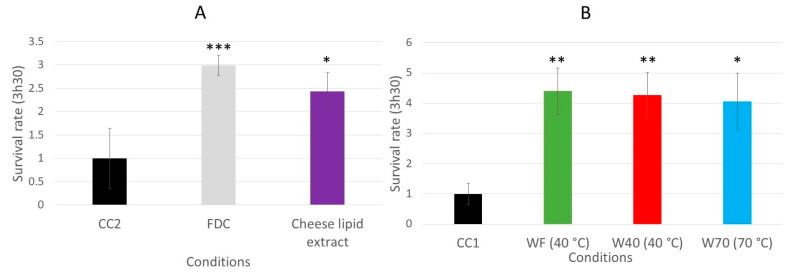
Relative survival rates of the wild-type *C. elegans* N2 strain on an oxidative medium after 5 days of incubation on a medium supplemented with FDC (freeze-dried cheese) and cheese-lipid extract (**A**) or aqueous extracts WF, W40 and W70 (**B**). The conditions were considered significantly different from the control when the *p*-value was lower than 0.05 (*), 0.01 (**), 0.001 (***) (Kruskal–Wallis test).

The impact of the dried cheese extracts (freeze-dried cheese (FDC), cheese-lipid extract, WF, W40 and W70) on the survival rate of the wild-type *C. elegans* N2 strain on an oxidative medium was evaluated (Figure 6). The survival rate was determined by measuring the worm viability after 3 h 30 min incubation on an agar H_2_O_2_-medium. The results demonstrated that the cheese extracts significantly influenced the ability of the worms to survive longer on the oxidative medium. Those incubated with the FDC and the cheese-lipid extract exhibited a better resistance to the oxidizing medium compared with the CC2 worms, with a survival rate of 3 (*p* = 0.0003) and 2.4 (*p* = 0.0306), respectively (Figure 6A). The same observation was made for the worms incubated with the cheese extracts WF, W40 and W70, where the survival rate increased to 4.4 (*p* = 0.0052), 4.3 (*p* = 0.0091) and 4.1 (*p* = 0.0181), respectively, compared with the CC1 condition (Figure 6B). The cheese extracts exhibited a beneficial effect on the worms by improving their survival rate on the oxidative medium.

### 3.4. Implication of the Signaling Pathways in the Survival of the C. elegans on the Oxidative Medium

**Figure 7:**
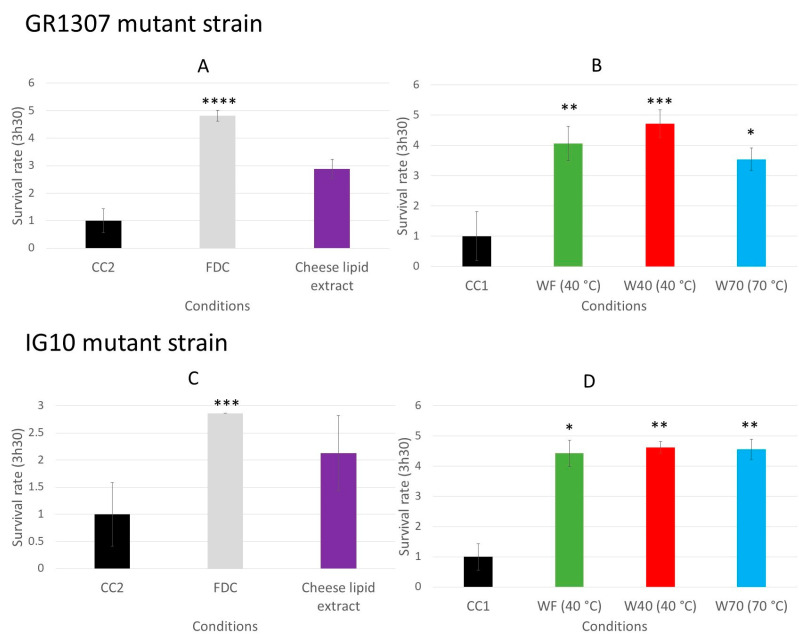
The relative survival rates of the *C. elegans* GR1307 strain (DAF-16 loss-of-function) (**A**,**B**) and IG10 strain (TOL-1 loss-of-function) (**C**,**D**) on an oxidative medium after 5 days of incubation on a medium supplemented with the cheese extracts (FDC (freeze-dried cheese), cheese-lipid extract and aqueous extracts WF, W40 and W70). The conditions were considered significantly different from the control when the *p*-value was lower than 0.05 (*), 0.01 (**), 0.001 (***) or 0.0001 (****) (Kruskal–Wallis test).

The increase of the survival rate of the worms, induced by the dried cheese extracts, may be due to an activation of the signaling pathways involved in the defense mechanisms of the worms, such as the insulin-like pathway or the p38 mitogen activated protein kinase (p38 MAPK) [8,9,22]. The same survival assay was performed with mutants to determine if these pathways are involved in the biological mechanism. The survival rate for the *C. elegans* GR1307 strain, which does not express *daf*-16, was determined by measuring the worm viability after 3 h 30 min of incubation on the oxidative medium. When incubated with the freeze-dried cheese (FDC) (*p* < 0.0001), and the cheese extracts WF (*p* = 0.0057), W40 (*p* = 0.0003) and W70 (*p* = 0.0371) (Figure 7A,B), the worms exhibited a significant resistance to the oxidative medium compared with that in their respective control conditions. The FDC increased the survival rate from 1 to 4.8. The worms incubated with WF, W40 and W70 had a survival rate between 3.5 and 4.7. The cheese-lipid extract tended to improve the worms’ resistance to the oxidative medium from 1 to 2.9. However, this increase was not significant compared with that in the CC2 group. The results suggested that only the cheese-lipid extract required the presence of the protein DAF-16 to improve the survival rate of the worms.

The same experiment was conducted with the *C. elegans* IG10 strain, which does not express the gene of the receptor *tol*-1 that is linked to the p38 MAPK signaling pathways involved in the nematode immunity. The absence of the receptor may prevent the activation of the pathway by the extracts and cancel the beneficial effects observed with the N2 strain. The same observations as for the GR1307 strain were made for the IG10 mutant. The worms incubated on the FDC or the dried aqueous extracts (W40, W70, WF) demonstrated a better resistance to the oxidizing medium (Figure 7C,D). The survival rate increased from 1 to 4.4 (*p* = 0.0162) for WF and 4.6 (*p* = 0.0037) for W40 and W70. For the FDC, it increased from 1 to 2.9 (*p* = 0.0005). Once again, the cheese-lipid extract tended to increase the worms’ resistance to the oxidative medium (survival rate of 2.1), but the effect observed was not significant in comparison with the CC2 condition. Based on these observations, only the cheese-lipid extract required the receptor TOL-1 in order to improve the survival rate of the worms.

### 3.5. Evaluation of the Expression of the Genes of Interest (GOI) daf-16, sek-1 and pmk-1

**Figure 8:**
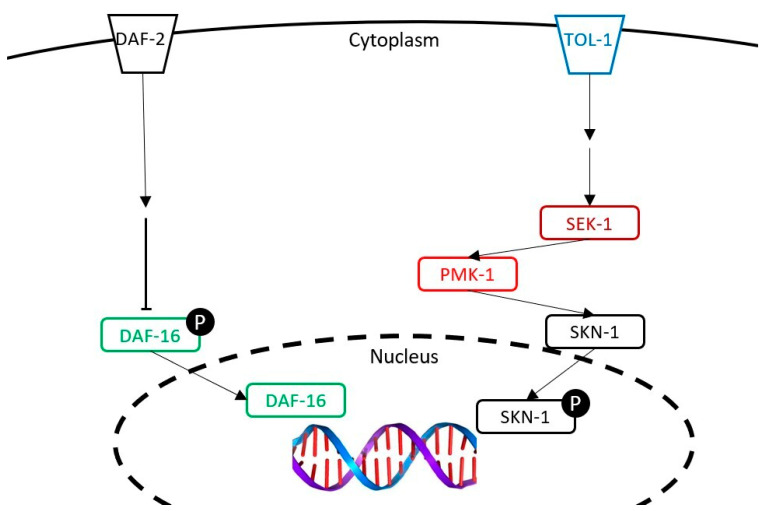
The representation of the insulin-like pathway and the p38 MAPK pathway studied in this article. The following genes were tested: *daf*-16 (studied with a mutant and a transcriptomic analysis), *tol*-1 (studied with a mutant), and *pmk*-1 and *sek*-1 (studied with a transcriptomic analysis).

The expression of the three genes of interest (*daf*-16, *sek*-1 and *pmk*-1) was investigated as two of these are implicated in the p38 MAPK pathway (Figure 8). The experiment was conducted during two different time periods: 3 days and 10 days of incubation with the dried cheese extracts. The freeze-dried cheese (FDC) and the cheese-lipid extract did not modulate the expression of any of the genes of interest after 3 days of incubation (Table 5). At 10 days, the FDC significantly upregulated the expression of the three genes to 2.78 for *daf*-16 (*p* = 0.0039), 2.89 for *sek*-1 (*p* = 0.0297) and 2.80 for *pmk*-1 (*p* = 0.016). The cheese-lipid extract upregulated the expression of *daf*-16 and *pmk*-1 to 3.41 (*p* = 0.0019) and 2.39 (*p* = 0.0094), respectively, after 10 days of incubation (Table 5).

With regard to the different dried aqueous extracts, at 3 days, only the extract WF overexpressed *daf*-16 up to 5.52 (*p* = 0.0018) before going back to a normal expression after 10 days. The expression of *sek*-1 and *pmk*-1 was not modified by the extract whatever the duration. As for W40, the results demonstrated that the extracts did not modulate the expression of the genes after 3 days of incubation (Table 6). At 10 days, W40 significantly increased the expression of *daf*-16 to 2.93 (*p* = 0.0043) and tended to overexpress *sek*-1 to 3.39. Finally, the extract W70 did not influence the expression of the three genes of interest. The results demonstrated that, except for W70, the extracts influenced the expression of *daf*-16. Concerning the genes *sek*-1 and *pmk*-1, only the FDC and the cheese-lipid extract were able to overexpress at least one of them.

### 3.6. Production of ROS in Human Blood Leukocytes Triggered by PMA

**Figure 9:**
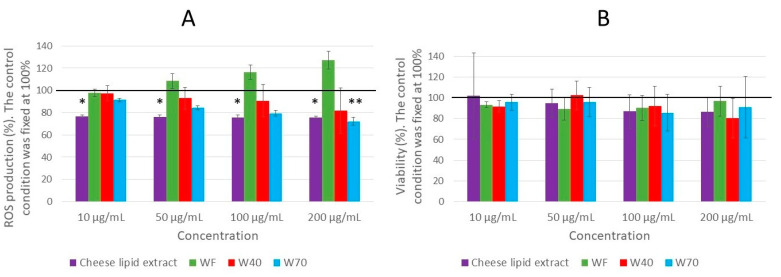
The effect of the cheese-lipid extract, WF, W40 and W70 on the ROS production in the leukocytes (**A**) and the viability of the leukocytes (**B**). The cells were treated with the indicated concentrations of the extract for 2 h, and measurements were made every 30 min. Data were expressed as relative production or viability in comparison with the control which was fixed at 100%. The conditions were considered significantly different from the control when the *p*-value was lower than 0.05 (*), 0.01 (**) (Kruskal–Wallis test).

The effect of the dried cheese extracts (cheese-lipid extract, WF, W40 and W70) on the production of the reactive oxygen species (ROS) was quantified in human blood leukocytes triggered by PMA (Figure 9A). Only two extracts exhibited the capacity to reduce the ROS production in the cells. A significant decrease was observed for W70 for the highest concentration (200 µg/mL), decreasing the ROS production by 28% (*p* = 0.0029). The other concentrations also tended to decrease the ROS production, but not significantly. Finally, the cheese-lipid extract significantly reduced the production of ROS for each concentration, with the same strength as no dose response was observed (by 23% for 10 µg/mL (*p* = 0.025) and by 24% for 50 µg/mL (*p* = 0.0202), 100 µg/mL and 200 µg/mL (*p* = 0.0124 for both concentrations)). The results obtained were not influenced by any toxic effects of the extracts, as no significant differences were observed with the leukocyte viability assay (Figure 9B).

### 3.7. Effect of the Dried Milk Extracts on the Longevity of Wild-Type C. elegans N2 Strain and Its Survival Rate on the Oxidative Medium

In order to determine if the milk could provide the same beneficial effect on the worms’ longevity as the cheese, a longevity assay was conducted with the dried milk extracts, using the wild-type *C. elegans* N2 strain. Indeed, the milk used for making the cheese could contain the same metabolites and so, could exert the same beneficial effect.

**Figure 10:**
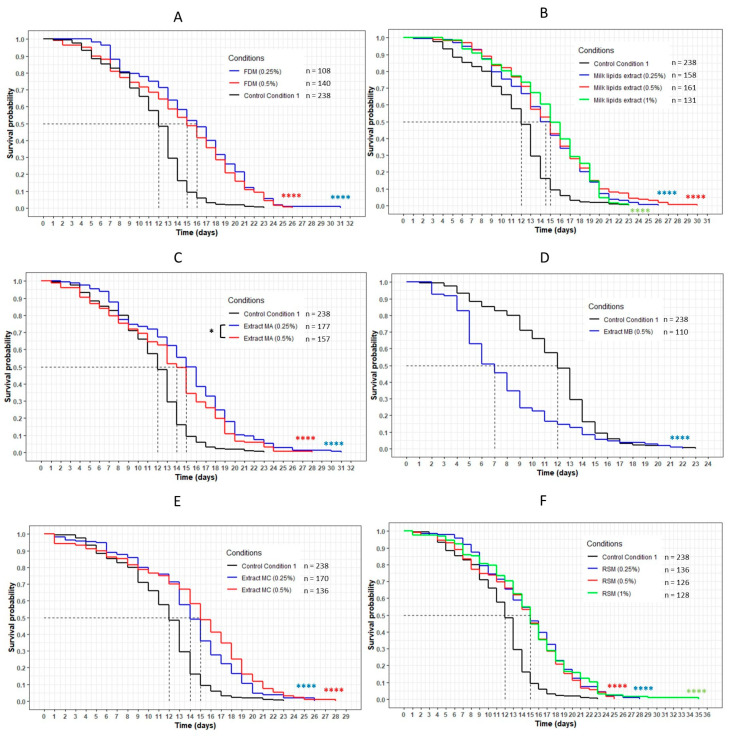
The influence of FDM (freeze-dried milk) (**A**), milk lipid extract (**B**), extracts MA (obtained with dichloromethane) (**C**), MB (obtained with ethyl acetate) (**D**), MC (obtained with ethanol) (**E**) and RSM (residual solid milk) (**F**) on the lifespan of wild-type *C. elegans* N2 strain. The worms were incubated on the medium supplemented with the dried extracts at day 0 and regularly fed with HK *E. coli* OP50. The conditions were considered significantly different when the *p*-value was lower than 0.05 (*) or 0.0001 (****). The asterisks next to the curves represent the differences with the control condition CC1. The asterisks next to the legend represent the differences between the extracts (log-rank test).

Incubating the worms with the freeze-dried milk (FDM) showed a significant increase in longevity for both concentrations tested (*p* < 0.0001) (Figure 10). The mean lifespan increased by 33% and 25% for the 0.25% and 0.5% concentrations, respectively (Table 7). The maximum lifespan also increased from 23 days (maximum lifespan of CC1) to 31 days (+35%) and 26 days (+13%), and the percentage of the population still alive when the worms on the CC1 condition had died was 6% and 5%, respectively. Moreover, no differences were observed between the two concentrations of FDM when compared to each other. The same observation was made for the milk lipid extract, the milk extracts MA (obtained with dichloromethane) and MC (obtained with ethanol) and the residual solid milk (RSM). Indeed, these conditions also significantly increased the lifespan of the worms (*p* < 0.0001). The mean lifespan increased between 21% and 25% for all conditions, and the percentage of the population still alive at 23 days reached a maximum of 5%. The maximum lifespan was more variable, with an increase of up to 52% with the RSM compared to the CC1 condition. Concerning the extract MA, a significant difference was observed between the two concentration curves (*p* = 0.04), with the 0.25% curve above the 0.5% concentration curve.

The extract MB, unlike the others, exhibited a significant negative effect on the lifespan of the wild-type *C. elegans* N2 strain. Indeed, the curve representing this extract was significantly below the CC1 curve (*p* < 0.0001) (Figure 10). The mean lifespan was reduced by 42% and the maximum lifespan was lower than the control, with a maximum of 22 days against 23 days (−4%) (Table 7).

**Figure 11:**
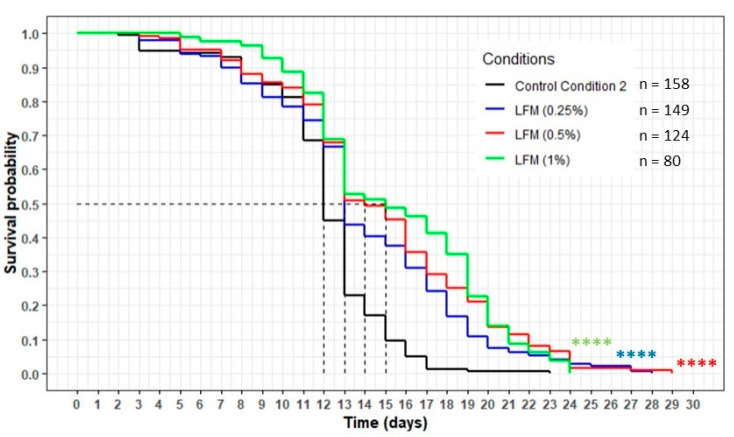
The influence of the LFM (lipid-free milk) on the lifespan of the wild-type *C. elegans* N2 strain. The worms were incubated on the medium supplemented with the dried extract at day 0 and regularly fed with HK *E. coli* OP50. The conditions were considered significantly different from the control conditions CC2 when the *p*-value was lower than 0.0001 (****) (log-rank test).

The worms incubated with the lipid-free milk (LFM) presented a significant increase in their lifespan compared with the CC2 condition, with the curves of each concentration of the extract above the CC2 curve (*p* < 0.0001) (Figure 11). The mean lifespan increased by 8%, 17% and 25% for the 0.25%, 0.5% and 1% concentrations, respectively (Table 8). The maximum lifespan increased between 4% and 26%, and the percentage of the population still alive when the worms on the CC2 condition had died was between 5% and 7%.

**Figure 12:**
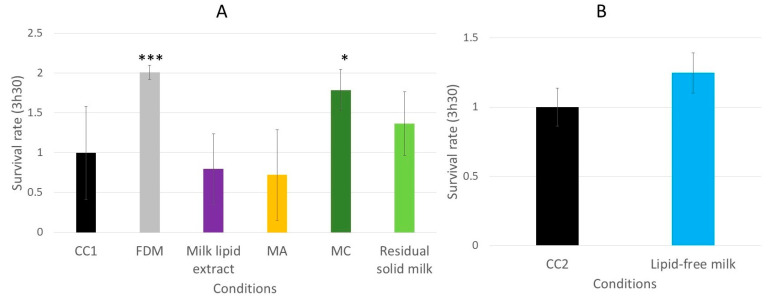
The relative survival rates of the wild-type *C. elegans* N2 strain on an oxidative medium after 5 days of incubation on a medium supplemented with FDM (freeze-dried milk), milk lipid extract, extract MA (obtained with dichloromethane), MC (obtained with ethanol) and RSM (residual solid milk) (**A**) or LFM (lipid-free milk) (**B**). The conditions were considered significantly different from the control when the *p*-value was lower than 0.05 (*) or 0.001 (***) (Kruskal–Wallis test).

The effects of the milk extracts on the survival abilities of the worms on the oxidative medium were also evaluated. Only the worms incubated with the FDM and the extract MC exhibited a better resistance to the oxidizing medium compared to the CC1 condition (Figure 12A), with a survival rate increasing from 1.0 for the CC1 to 2.0 (*p* = 0.0006) and 1.8 (*p* = 0.0142), respectively. The other extracts did not show any significant effect on the survival of the worms.

With the exception of the extract MB, the results of our lifespan assay demonstrated that all of the other milk extracts (FDM, milk lipid extract, MA, MC, RSM and LFM) exerted a beneficial effect and were able to significantly increase the lifespan of the worms. However, in the survival assay on the oxidative medium, the effects of these extracts, still excepting MB, on the worms’ survival rate were not as noteworthy as those of the cheese extracts (FDC, cheese-lipid extract, WF, W40, W70). Indeed, only two milk extracts (FDM and MC) exhibited an effect on the worms.

## 4. Discussion

### 4.1. The Transcription Factor DAF-16 Is Implicated in the Mechanism by Which the Dried Cheese Extracts Increase Longevity

In our previous study, we demonstrated the beneficial effects of different dried cheese extracts (the freeze-dried cheese (FDC), the cheese-lipid extract and the three cheese extracts obtained with water) on nematode longevity [4]. To better understand the mechanisms of action, the present study was focused on the identification of the signaling pathways that may be involved in these beneficial effects. *C. elegans* has already been the subject of many investigations and is now well described [23]. The insulin-like pathway, also named DAF-2/DAF-16, has been reported to be involved in the development and in the longevity phenomenon of the worm [11,24]. Many studies have highlighted that the transcription factor DAF-16 regulates the lifespan by influencing downstream genes [10,11]. As our extracts were shown to increase longevity, an assumption was established that DAF-16 was associated with this phenomenon. Two experiments were performed to determine the involvement of DAF-16: a lifespan assay with the GR1307 mutant strain that does not express DAF-16 and the study of the cellular localization of DAF-16 with the transgenic strain TJ356 expressing DAF-16::GFP. These assays were completed with a transcriptomic analysis to determine the expression of *daf*-16 when the worms were incubated with the extracts.

It has been shown that the FDC significantly increased the lifespan of the wild-type *C. elegans* N2 strain. The longevity assay on the DAF-16 loss-of-function strain (GR1307) showed a difference in the effect of the cheese, with a disappearance of the beneficial effects. There was no variation in the mean and maximum lifespan of the worms incubated with FDC compared with the CC2 worms. The results demonstrated that FDC cannot increase longevity without DAF-16. However, for both concentrations, the FDC curve was significantly above the CC2 curve, suggesting that the FDC exercised a beneficial effect on the worms, allowing a larger part of the population to remain alive for a given amount of time compared with the CC2 condition. The hypothesis of the implication of DAF-16 was also consolidated by the observations of the cellular localization of DAF-16::GFP (Figure 5) and the analysis of gene expression. Both experiments validated the implication of DAF-16. The FDC tended to translocate the transcription factor into the nuclei after 2 h of incubation. The gene expression analysis showed that the FDC significantly upregulated the expression of *daf*-16 at 10 days. All results validated the implication of the DAF-16 transcription factor in the mechanisms of action of the FDC in increasing the lifespan of the nematode.

The cheese-lipid extract presented a dose effect during the longevity assay. A significant increase in longevity was only observed for the 0.5% (*p* = 0.02). The study of the DAF-16::GFP consolidated the observation made during the longevity assay, as well as the RT-qPCR analysis, for which the extract significantly overexpressed *daf*-16 at 10 days. These results validated the implication of the transcription factor DAF-16 in the mechanisms of action of the extract in increasing the longevity in the wild-type *C. elegans* N2 strain.

The monitoring of the cellular localization of DAF-16::GFP showed that the extracts W40 and WF tended to activate the signaling pathway by translocating the protein into the nuclei. The transcriptomic analysis consolidated this observation for WF and W40 which significantly up-regulated the expression of the gene at 3 days and at 10 days, respectively. These observations were reinforced by the inability of the extracts to increase the longevity of the GR1307 strain (DAF-16 loss-of-function). Even more, the curves representing the extracts were significantly below the CC1 curve (for both concentrations), suggesting that the transcription factor is necessary and required for WF and W40 to exhibit a beneficial effect. Concerning W70, no differences were observed with the CC1 condition for either concentration. Although W70 did not overexpress the gene *daf*-16, it tended to translocate the transcription factor into the nuclei. In agreement with the observation made on the wild-type *C. elegans* N2 strain the extract did not show any toxic effect on the worms. All the results obtained validated the hypothesis that the DAF-16 transcription factor is involved in the biological mechanism, allowing the longevity of the nematode to increase when incubated with the extracts. The proof of the implication of DAF-16 completes the results obtained in the previous study [4], by characterizing the mechanisms of action of the extract.

As DAF-2/DAF-16 pathway is a homologue of the insulin pathway in human, it is conceivable that the effects of the bioactive extracts on the *C. elegans* model may be similar in humans, using the same pathway. Further studies are required to investigate this hypothesis.

### 4.2. The Dried Cheese Extracts Influenced the Survival of the Worms on the Oxidative Medium

During aging, the defense mechanisms of the worms are weakened, making the nematode more sensitive to the oxidative stress [9]. The beneficial effects of the dried cheese extracts observed in longevity may be due to an improvement of the worms’ resistance to the oxidative stress and/or to the interaction with the DAF-16 transcription factor. To test this assumption, the effect of the extracts on the survival rate of the worms on the oxidative medium was evaluated.

The assay demonstrated that the worms incubated with the dried cheese extracts exhibited a better resistance to the oxidative medium, with an increase in the survival rate of up to 4.4 (Figure 6). Following these observations, the investigation of the signaling pathways implicated in the mechanisms of action was performed with the GR1307 (which does not express DAF-16) and IG10 (which does not express the receptor TOL-1) mutant strains. The insulin-like pathway is also described to be involved in immunity and stress resistance [22,25]. The results of the longevity assays suggested that DAF-16 is implicated in the beneficial effect of the extracts. The same survival assay on an oxidative medium was performed with the GR1307 strain. In absence of the protein DAF-16, no variation in the effect of the cheese extracts was observed in the worms, as evidenced by the survival rates, except for the cheese-lipid extract for which the increased survival rate was not significant compared with the control. This extract requires the expression of the protein DAF-16 to exhibit an improvement in the worms’ resistance to the oxidative stress.

Another signaling pathway was investigated to describe the mechanisms of action of the cheese extracts. The p38 MAPK is a pathway involved in the human immune system [26]. This pathway is highly conserved in *C. elegans*, and is also implicated in the worms’ resistance to oxidative stress by the synthesis of glutathione [8,22,27]. The receptor TOL-1, which is connected to the p38 MAPK, is a homolog of the toll-like receptor family in humans, which is implicated in immunity [9] and may play a role in the effect of the extracts. The survival assay on the oxidative medium performed with the TOL-1-deficient mutant strain showed the same results as the GR1307 strain. Indeed, the lipid extract alone could not significantly increase the survival rate of the worms without the presence of TOL-1, suggesting that the lipid extract also needs the p38 MAPK as well to exert its effect. The transcriptomic analysis revealed that the expression of two downstream genes of the p38 MAPK pathway, *sek*-1 and *pmk*-1, was modulated by some cheese extracts. The FDC significantly upregulated *sek*-1 and *pmk*-1, and the cheese-lipid extract, only influenced the expression of *pmk*-1, suggesting that the two extracts exert an effect via the p38 MAPK signaling pathway. These results suggest that the cheese-lipid extract requires the activation of the two signaling pathways to improve the resistance of the worms. With regard to WF, W40 and W70, they did not influence the expression of these genes. The results from the assays suggest that they may use another pathway for increasing the survival rate of the worms. Further mechanistic studies are needed to better understand their mechanisms of action.

### 4.3. Influence of the Dried Cheese Extracts on ROS Production in Human Leukocytes

The FDC (freeze-dried cheese), the cheese-lipid extract and the cheese extracts WF, W40 and W70 demonstrated the ability to increase the longevity of the wild-type *C. elegans* N2 strain, as well as its survival rate on an oxidative medium, suggesting an action on the worms’ resistance to the oxidative stress. In keeping with the investigation of the effects of the extract on this stress, an in vitro assay was conducted to determine the capacity of four extracts (cheese-lipid extract, WF, W40 and W70) to reduce the ROS production in human leukocytes. Only two extracts reduced the amount of ROS produced by the cells when triggered with PMA. The cheese-lipid extract seemed to exhibit an effect without any dose response, suggesting that the maximum efficient concentration was reached. Further studies with lower concentrations should give more information on the activity of the cheese-lipid extract on the ROS production. Conversely, the extract W70 only demonstrated a beneficial effect for the highest concentration (200 µg/mL). For the lower concentrations, the extract tended to decrease the ROS production but without significant differences, suggesting that the effect of W70 increased with the concentration. This assay allowed us to make the correlation between the findings observed in *C. elegans* and in humans. The two effective extracts were able to improve the resistance of the worms to the oxidative medium and to reduce the ROS production in the human lymphocyte cells. Further studies are required in order to determine if the mechanisms of action involved in human cells are similar to those in *C. elegans*.

### 4.4. The Milk and Its Extracts Exert a Lower Beneficial Effect on the Wild-Type C. elegans N2 Strain Compared with the Cheese and Its Extracts

The goal of this assay was to determine if the milk could exert the same effect as the cheese on the longevity and survival rate of the *C. elegans*. The results suggested that, except for the extract MB (obtained with ethyl acetate as a solvent), all of the dried milk extracts significantly increased the longevity of the wild-type *C. elegans* N2 strain. However, this beneficial effect seems lower in comparison with the effect of the dried cheese extracts as determined in our previous study [4]. Indeed, the comparison between the milk extracts and the equivalent cheese extracts, at the same concentration, revealed that milk was less efficient in improving the longevity of the nematode *C. elegans* than the cheese. The increase of the mean lifespan was higher with the freeze-dried milk (FDM) at 0.5% than the freeze-dried cheese (FDC) at 0.5% (+25% for the FDM and +18% for the FDC), but the maximum lifespan was lower. The FDM increased the maximum lifespan by 13%, whereas the FDC increased it by 63% at the same concentration. Moreover, the part of population still alive on the conditions, when the worms of the control had died, was higher with the FDC, with a maximum of 13% of the population still alive, whereas only a maximum of 5% was observed with the worms on the FDM. The same observation was made when the cheese-lipid extract was compared to the milk-lipid extract: a mean lifespan higher with the milk-lipid extract (+30% against +18%, for the same 0.5% concentration) but the maximum lifespan was lower than the cheese-lipid extract (+30% against +37%, for the same 0.5% concentration). The differences observed in their biological response may be due to a variation in the metabolite composition and/or concentration between these two dairy foods, induced by the microorganism’s activities (fermentation, etc.) of the milk during the cheese-making process. This observation was confirmed by the fact that the cheese extracts A (extracted with dichloromethane) and B (extracted with ethyl acetate) killed the worms instantly, whereas survival curves from the longevity study with the equivalent extracts of milk (MA and MB) were recovered, confirming the variation in the metabolite composition, or their concentration, between the two dairy foods.

The same observations were noted in the survival assay on the oxidative medium. The results suggested that the milk extracts were less beneficial than the cheese extracts. Indeed, only two milk extracts (freeze-dried milk (FDM) and extract MC) improved the survival of the worms, whereas the five cheese extracts (FDC, cheese-lipid extract, WF, W40 and W70) exerted a beneficial effect on the wild-type *C. elegans* N2 strain. Moreover, the survival rate was higher for the worms incubated with the cheese extracts. The survival assay on the oxidative medium, as well as the longevity assay, validated that the milk extracts had a less favorable impact on the *C. elegans* nematode.

## 5. Conclusions

This study allowed us to deepen the understanding of the biological effects of dried cheese extracts on longevity and resistance to oxidative stress in *C. elegans*. To our knowledge, this investigation had never been performed. The study of the signaling pathways involved in the mechanisms of action of the cheese extracts revealed, for the first time, that the insulin-like pathway is implicated, via DAF-16, to increase longevity. The extracts also revealed the capacity to increase the survival rate of the worms that were incubated on an oxidative medium. However, no evidence of the implication of the DAF-2/DAF-16 and/or the p38 MAPK in this mechanism has been highlighted. Only the cheese-lipid extract seemed to require these two pathways to improve the worms’ resistance. The beneficial effects of the cheese extracts on the wild-type *C. elegans* N2 strain were correlated with their effect in human leukocytes. Indeed, two extracts (cheese-lipid extract and W70) decreased the ROS production, confirming the link between the results obtained with the nematode and human cells. These results allow us to hypothesis that the benefits of the raw-milk cheese could be similar in the aging process in humans. However, further studies on human cells are needed to pursue the investigation of the action of the extracts and understand the mechanisms implied in this phenomenon. Finally, the comparison of the effects of the milk and cheese extracts on longevity and the survival rate of the worms demonstrated a less favorable effect of the milk compared with the cheese, suggesting that the bioactive metabolites were only present, or were at a higher concentration, in the fermented food. The investigation of the bioactive metabolites in the goat cheese should continue, by subfractionating the interesting extracts and evaluating the biological effects of the resulting subfractions on the *C. elegans* and human cells in order to deepen our knowledge of the biological activity of the cheese. Alongside, a comparison of the beneficial effects of the goat cheese from this study and other raw-milk cheeses should give more information regarding the bioactive metabolites in cheese and its potential applications concerning human health.

## Figures and Tables

**Table 1:** Concentrations of the dried milk extracts used for supplementing the medium. Concentrations are expressed in percentage of extracts relative to the volume of medium.

Extracts	Concentration (*w*/*v*)		
	0.25%	0.5%	1%
Freeze-dried milk (FDM)	X	X	
Milk lipid extract (ML)	X	X	X
Lipid-free milk (LFM)	X	X	X
Extract MA	X	X	
Extract MB		X	
Extract MC	X	X	
Residual solid milk (RSM)	X	X	X

**Table 2:** Targeted *C. elegans* genes primers for qPCR analysis.

Gene Name	Gene Type	Forward Primer (5′-3′)	Reverse Primer (5′-3′)	Reference
Y45F10D.4	housekeeping	CGAGAACCCGCGAAATGTCGGA	CGGTTGCCAGGGAAGATGAGGC	[19]
*daf-16*	GOI	TTCAATGCAAGGAGCATTTG	AGCTGGAGAAACACGAGACG	[19,21]
*sek-1*	GOI	GCCGATGGAAAGTGGTTTTA	TAAACGGCATCGCCAATAAT	[19,21]
*pmk-1*	GOI	CCGACTCCACGAGAAGGATA	AGCGAGTACATTCAGCAGCA	[19,21]

**Table 3:** Data of the longevity assay of the *C. elegans* GR1307 strain on the medium supplemented with the FDC (freezz-dried cheese) and the cheese-lipid extract, and CC2 as control. Mean lifespan, maximum lifespan and the percentage of population still alive were taken from the survival curves in Figure 2. *p*-values were calculated by comparing conditions with CC2 using the log-rank test.

Tested Conditions	Concentration (*w*/*v*) (%)	Mean Lifespan (Days)	Maximum Lifespan (Days)	Relative Increase in the Maximum Lifespan (%)	Percentage of Population Still Alive at 17 Days (%)	*p*-Value
**CC2**	-	10	17	-	0	-
**Freeze-Dried Cheese (FDC)**	0.5	10	17	0	0	0.001
	1	10	17	0	0	0.03
**Cheese-lipid extract**	0.5	10	18	+6	1	0.02
	1	10	16	−6	0	0.5

**Table 4:** Data of the longevity assay of the *C. elegans* GR1307 strain on the medium supplemented with the aqueous extracts WF, W40 and W70, and CC1 as a control. Mean lifespan, maximum lifespan and the percentage of population still alive were taken from the survival curves in Figure 3. *p*-values were calculated by comparing conditions with CC1 using the log-rank test.

Tested Conditions	Concentration (*w*/*v*) (%)	Mean Lifespan (Days)	Maximum Lifespan (Days)	Relative Increase in the Maximum Lifespan (%)	Percentage of Population Still Alive at 17 Days (%)	*p*-Value
CC1	-	9	17	-	0	-
Extract WF (40 °C)	0.5	7	19	+12	1	<0.0001
	1	8	19	+12	2	0.0008
Extract W40 (40 °C)	0.5	8	19	+12	2	<0.0001
	1	8	20	+18	1	0.0002
Extract W70 (70 °C)	0.5	10	22	+29	2	0.9
	1	9	22	+29	5	0.09

**Table 5:** The relative expression of the three *C. elegans* genes of interest after 3 days or 10 days of incubation on a medium supplemented with FDC (freeze-dried cheese) or cheese-lipid extract in comparison with CC2 condition. The expressions were considered significantly different when the *p*-value was lower than 0.05 (*) or 0.01 (**), and simultaneous when the expression change was of at least 2 times higher or 0.5 times lower.

	Genes of Interest		
Conditions	*daf-*16	*sek-*1	*pmk-*1
FDC 3 days	1.95	1.13	1.00
FDC 10 days	2.78 **	2.89 *	2.80 *
Cheese-lipid extract 3 days	1.07	1.02	1.09
Cheese-lipid extract 10 days	3.41 **	1.19	2.39 **

**Table 6:** The relative expression of the three *C. elegans* genes of interest after 3 days or 10 days of incubation on a medium supplemented with aqueous extracts WF, W40 or W70, in comparison with CC1 condition. The expressions were considered significantly different when the *p*-value was lower than 0.01 (**), and simultaneous when the expression change was of at least 2 times higher or 0.5 times lower.

	Genes of Interest		
Conditions	*daf-*16	*sek-*1	*pmk-*1
WF (40 °C) 3 days	5.52 **	1.11	1.27
WF (40 °C) 10 days	1.00	1.87	1.01
W40 (40 °C) 3 days	1.08	0.83	1.24
W40 (40 °C) 10 days	2.93 **	3.39	0.67
W70 (70 °C) 3 days	1.20	0.80	1.08
W70 (70 °C) 10 days	1.96	1.26	0.59

**Table 7:** Data of the longevity assay of the wild-type *C. elegans* N2 strain on the medium supplemented with the FDM (freeze-dried milk), milk lipid extract, extracts MA (obtained with dichloromethane), MB (obtained with ethyl acetate), MC (obtained with absolute ethanol) and the RSM (residual solid milk), and CC1 as a control. Mean lifespan, maximum lifespan and the percentage of the population still alive are from the survival curves. *p*-values were calculated by comparing conditions with the CC1 using the log-rank test.

Tested Conditions	Concentration (*w*/*v*) (%)	Mean Lifespan (Days)	Maximum Lifespan (Days)	Relative Increase of the Maximum Lifespan (%)	Percentage of Population Still Alive at 23 Days (%)	*p*-Value
**CC1**	-	12	23	-	0	-
**Freeze-Dried Milk (FDM)**	0.25	16	31	+35	6	<0.0001
	0.5	15	26	+13	5	<0.0001
**Milk Lipid Extract**	0.25	14.5	26	+13	3	<0.0001
	0.5	15	30	+30	5	<0.0001
	1	15	23	0	0	<0.0001
**Extract MA**	0.25	15	31	+35	5	<0.0001
	0.5	14	28	+22	3	<0.0001
**Extract MB**	0.5	7	22	−4	0	<0.0001
**Extract MC**	0.25	14	26	+13	2	<0.0001
	0.5	15	28	+22	4	<0.0001
**Residual Solid Milk (RSM)**	0.25	15	28	+22	5	<0.0001
	0.5	15	25	+9	5	<0.0001
	1	15	35	+52	4	<0.0001

**Table 8:** Data of the longevity assay of the wild-type *C. elegans* N2 strain on the medium supplemented with the LFM (lipid-free milk) and CC2 as a control. Mean lifespan, maximum lifespan and the percentage of population still alive are from survival curves. *p*-values were calculated by comparing conditions with CC2 using the log-rank test.

Tested Conditions	Concentration (*w*/*v*) (%)	Mean Lifespan (Days)	Maximum Lifespan (Days)	Relative Increase of the Maximum Lifespan (%)	Percentage of Population Still Alive at 23 Days (%)	*p*-Value
CC2	-	12	23	-	0	-
Lipid-Free Milk (LFM)	0.25	13	28	+22	5	<0.0001
	0.5	14	29	+26	7	<0.0001
	1	15	24	+4	5	<0.0001

## Data Availability

All relevant data are within this manuscript.
